# Protective Effect of Yi Shen Pai Du Formula against Diabetic Kidney Injury via Inhibition of Oxidative Stress, Inflammation, and Epithelial-to-Mesenchymal Transition in *db/db* Mice

**DOI:** 10.1155/2021/7958021

**Published:** 2021-08-31

**Authors:** Qilin Zhang, Xiaocui Liu, Mitchell A. Sullivan, Chen Shi, Bin Deng

**Affiliations:** ^1^Department of Pharmacy, Union Hospital, Tongji Medical College, Huazhong University of Science and Technology, 430030 Wuhan, China; ^2^Hubei Province Clinical Research Center for Precision Medicine for Critical Illness, 430030 Wuhan, China; ^3^Department of Pharmacy, Taihe Hospital, Hubei University of Medicine, No. 32 South Renmin Road, Huibei, Shiyan 442000, China; ^4^Glycation and Diabetes Group, Mater Research Institute-the University of Queensland, Translational Research Institute, Brisbane, Queensland 4072, Australia

## Abstract

**Objective:**

Diabetic kidney disease (DKD) is one of the most common chronic microvascular complications of diabetes; however, there remains a lack of effective therapeutic strategies. Yi Shen Pai Du Formula (YSPDF), a traditional Chinese medicine preparation, has been clinically used in treating chronic kidney disease (CKD) for more than 20 years. However, whether YSPDF has a therapeutic effect on DKD has not been studied.

**Methods:**

This study was conducted to investigate the effect of YSPDF administration on *db/db* mice, a model of type 2 diabetes that develops DKD, and reveal its underlying mechanism of action through a high glucose- (HG-) induced renal injury cell model.

**Results:**

We found that YSPDF significantly improved numerous biochemical parameters (fasting blood glucose, serum creatinine, blood urea nitrogen, 24 h urine total protein, total cholesterol, and total triglycerides) and ameliorated the abnormal histology and fibrosis of renal tissue. Moreover, the status of oxidative stress and levels of inflammatory cytokines (TNF-*α*, IL-6, IL-1*β*, and MCP-1) were markedly inhibited by YSPDF treatment. YSPDF treatment significantly mitigated renal fibrosis, with evidence suggesting that this was by inhibiting epithelial-to-mesenchymal transition (EMT) via suppression of the TGF-*β*1/Smad pathway. Interestingly, the expression of Nrf2, HO-1, and NQO1, proteins known to be associated with oxidative stress, were significantly increased upon administration of YSPDF. The levels of NLRP3 inflammasome proteins, including NLRP3, ASC, caspase-1, and cleaved caspase-1 were decreased in the YSPDF-treated group. Cell experiments showed that YSPDF inhibited EMT and the NLRP3 inflammasome in HG-exposed HK-2 cells, possibly via activation of Nrf2.

**Conclusion:**

Our study indicates that YSPDF may ameliorate renal damage in *db/db* mice via inhibition of oxidative stress, inflammation, and EMT, with the mechanism potentially being related to the activation of the Nrf2 pathway.

## 1. Introduction

Diabetic kidney disease (DKD), also known as diabetic nephropathy (DN), is one of the most common chronic microvascular complications of diabetes and is the leading cause of end-stage renal failure (ESRD). The growing prevalence of diabetes has resulted in a rapid increase in the global incidence of DKD, bringing an enormous economic burden to society [1, 2]. The pathogenesis of DKD has not been fully elucidated, and currently, there are no effective treatments [3]. Thus, developing highly effective and low-toxic drugs that can prevent the occurrence and progression of DKD has become a serious medical challenge.

Tubulointerstitial fibrosis is a crucial pathological alteration underlying the progression of DKD [4, 5]. Epithelial-to-mesenchymal transition (EMT), characterized by a loss of epithelial phenotype and a gain of profibrotic features, plays a crucial role in the development and progression of tubulointerstitial fibrosis [4]. Accumulating evidence indicates that EMT accelerates the generation of renal fibrosis in DKD. When EMT is activated, the kidney fibroblasts have been shown to translate into myofibroblasts, secreting excessive extracellular matrix (ECM) and eventually causing the development of fibrosis [5]. EMT is regulated by many factors. It has been demonstrated that transforming growth factor-*β*1 (TGF-*β*1) is able to induce EMT activation in renal tubular epithelial cells [6, 7]. TGF-*β*1 phosphorylates downstream proteins Smad2 and Smad3, increasing the expression level of *α*-smooth muscle actin (*α*-SMA) and decreasing the expression level of E-cadherin [3]. Therefore, inhibiting EMT via the TGF-*β*1/Smad pathway might be an effective therapeutic target for DKD.

It has been widely reported that oxidative stress and an inflammatory response are implicated in DKD [8, 9]. High glucose not only promotes generation of reactive oxygen species (ROS) by inhibiting the nuclear factor-erythroid 2-related factor 2 (Nrf2) pathway but also activates the nucleotide binding and oligomerization domain-like receptor family pyrin domain-containing 3 (NLRP3) [10–12]. Key proteins of the Nrf2 pathway, Nrf2, HO-1, and NQO1, have been shown to be significantly reduced in DKD mice, indicating that the Nrf2 pathway may protect the kidney from damage by decreasing oxidative stress [13]. When the NLRP3 inflammasome is activated, the secretion of proinflammatory cytokines IL-18 and IL-1*β* is increased, with kidney inflammation and fibrosis being amplified [14]. A knockout of NLRP3 significantly attenuated the inflammatory response of renal tissue and improved renal function in a DKD mouse model [15]. Moreover, it has been reported that the renal NLRP3 inflammasome can also be activated by elevating levels of ROS [16]. In summary, reduction of oxidative stress and inflammation could be effective approaches to attenuate kidney injury induced by hyperglycemia.

*Astragali radix* (Huang Qi) and *Rhei radix et rhizome* (Da Huang) is a classical formula for treating kidney injury [17]. YSPDF is an innovative Chinese medicine prescribed on the basis of containing *Astragali radix* and *Rhei radix et rhizome*. It also contains two additional traditional Chinese medicines: *Hirudo* (Shui Zhi) and *Bombyx batrytocatus* (Jiang Can). The pharmacological effect of YSPDF on chronic kidney disease (CKD) has been systematically studied; however, whether YSPDF has a therapeutic effect on DKD has not been investigated [18]. In the prescription of YSPDF, *Astragali radix* is a “Jun medicine” with the traditionally described function of replenishing qi. It has been reported that the nephritis prescription composed of *Astragalus membranaceus* and *Angelica sinensis* could significantly reduce urinary albumin and serum creatinine hydration in DN rats and protect renal function [19]. *Rhei radix* is a “Chen medicine” with the traditionally described effect of cleaning away heat, purgation, and detoxification. Modern pharmacology studies have shown that the extract of *Rhei radix* could effectively inhibit renal oxidative stress, lipid peroxidation, and ECM accumulation to reduce diabetic kidney damage [20, 21]. *Hirudo* and *Bombyx batrytocatus* were the adjuvant medicine, whose function were promoting blood circulation to remove blood stasis and eliminating wind. Studies have shown that hirudin in *Hirudo* has effects as an anticoagulant and antithrombotic, which can inhibit renal fibrosis by reducing the levels of renal inflammatory factors such as IL-1 and IL-6 and renal tubular EMT [22]. Thus, according to the current literature, we propose a hypothesis that YSPDF may have a positive effect on the treatment of DKD, and its mechanism may be related to inhibiting renal EMT, oxidative stress, and inflammation.

In the present study, we systematically investigated the protective effect of YSPDF on diabetic kidney injury in *db/db* mice, aiming to reveal potential mechanisms. This work will provide new knowledge on the pharmacological effects of YSPDF and help determine if YSPDF could be used as a clinical therapy for DKD.

## 2. Materials and Methods

### 2.1. Preparation of Yi Shen Pai Du Formula (YSPDF)

YSPDF was prepared using extractions from four traditional Chinese medicines: *Astragali radix* (Huang Qi) (lot no. 20170516), *Rhei radix et rhizome* (Da Huang) (lot no. 20170411), *Hirudo* (Shui Zhi) (lot no. 20170520), and *Bombyx batrytocatus* (Jiang Can) (lot no. 20170510) (Table 1). All the botanical and animal names are recorded and can be validated using http://mpns.kew.org/mpns-portal/?_ga=1.111763972.1427522246.1459077346. Crude YSPDF was purchased from Bozhou Jinshaotang Chinese Medicine Decoction Co., Ltd. The ratio of *Astragali radix*, *Rhei radix et rhizome*, *Hirudo*, and *Bombyx batrytocatus* in the formula was 25 : 5 : 3 : 3. This crude YSPDF was powdered and subjected to reflux extraction with 10 volumes of water for 1.5 h. The aqueous extracts were filtered and collected. The extraction was repeated twice with the method described above, and then, all the extracts were evaporated to dryness under reduced pressure. Finally, 1 g of YSPDF extracts were equivalent to 1.44 g of original crude material. The pulverized YSPDF extracts were dispersed and dissolved in distilled water for animal experiments.

The quality of the extracted YSPDF was analyzed using reversed phase high-performance liquid chromatography (RP-HPLC) on the basis of a method established by the Department of Pharmacy, Union Hospital, Tongji Medical College, Huazhong University of Science and Technology (Wuhan, China) [23]. The HPLC chromatogram was obtained from the HPLC unit (Agilent 1260, USA) and Agilent TC-C18 column (4.6 mm × 250 mm, 5 *μ*m particle size) with the temperature being maintained at 30°C. The mobile phase consisted of solutions with varying ratios of A (acetonitrile) and B (0.2% formic acid) at a flow rate of 1.0 mL/min and the detection wavelength was at 260 nm. A linear gradient elution was performed with the gradient procedure as follows: 0-20 min, 20%-40% A; 20-30 min, 40% A; 30-35 min, 90% A; 35-40 min, 20% A. A standard was created that contained the following: calycosin glucoside, aloe-emodin, rhein, emodin, chrysophanol, and physcion.

### 2.2. Animals Experiment

Male mice on a C57BL/6JNju background (8 weeks) were purchased from the Hubei Provincial Center for Food and Drug Safety. Male mice on C57BL/BKS-Lepr^em2Cd479^/Nju (C57BL/6JNju-*db/db*, genotyping is (Lepr^db^) mut/mut) background (8 weeks) were purchased from the Model Animal Research Center of Nanjing University. Mice were bred in an SPF room with standard cages (4 mice/cage) under the following conditions: 22 ± 1°C and a 12 h dark-light cycle. All animals had ad libitum access to water and standard chow (6% kcal from fat, 14.3 MJ kg^−1^, Hubei Provincial Center for Disease Control and Prevention). All animal experiments were approved (no. 2019S938) by the institutional Animal Care and Use Committee of Tongji Medical College, Huazhong University of Science and Technology. The animal care and experimental procedures were carried out in accordance with the Guidelines of the Institutional Animal Care and Use Committee of Tongji Medical College and the National Institutes of Health Guide for the Care and Use of Laboratory Animals.

After one week acclimatization, 10 healthy C57BL/6 mice and 20 *db/db* mice were divided into 3 groups: Group I (NC): healthy mice, Group II (DKD): *db/db* mice, Group III (DKD+YSPDF): *db/db* mice were administered intragastrically with 0.4 mL of YSPDF (2 g/kg) once daily. The usage of YSPDF in this study was determined based on the clinical usage of YSPDF and previous animal study [24]. Mice in the NC and DKD groups were administered intragastrically with an equal volume of distilled water. Drug treatment lasted for 8 weeks until the sacrifice of the mice.

### 2.3. Sample Collection and Metabolic Index Detection

Before the mice were sacrificed, they were placed in metabolic cages for 1 day. Urine samples were collected over the 24 hours for the analysis of urinary creatinine (UCr) and 24 h urine total protein (24 h UTP). At the end of the experiment, the mice were fasted overnight and anaesthetized with sodium pentobarbitone (150 mg/kg in traperitoneal). The body weight, liver weight, kidney weight, and fasting blood glucose level (FBG) were measured. The FBG levels were measured by a glucometer 12 h after fasting at the end of the study. Blood samples of each mouse were collected and immediately separated by centrifugation (1200 g, 4°C, 15 min) to obtain the serum. The serum samples were kept for the test of serum creatinine (SCr), blood urea nitrogen (BUN), total cholesterol (TC), total triglycerides (TG), high-density lipoprotein (HDL), and low-density lipoprotein (LDL). The kidney tissues were collected and divided into two parts across the sagittal plane: one part was fixed in 10% formalin and the rest was stored at -80°C.

### 2.4. Biochemical Analysis

The urinary creatinine (UCr) and SCr were measured by a creatinine assay kit (C011-2-1). The 24 h urine total protein (24 h UTP) was detected by a urine protein test kit (C035-1-1). The level of BUN (C013-2-1), TG (C110-1-1), TC (C111-1-1), LDL (A113-1-1), and HDL (A112-1-1) in the serum, and the level of ROS (E004-1-1), glutathione (GSH, A006-2-1), malondialdehyde (MDA, A003-1-2), superoxide dismutase (SOD, A001-3-2), and catalase (CAT, A007-1-1) in the kidney tissues were determined by ELISA kits (Nanjing Jian Cheng Bioengineering Institute, China), respectively. The content of interleukin-1*β* (IL-1*β*, JL18442), interleukin-6 (IL-6, JL20268), tumor necrosis factor-*α* (TNF-*α*, JL10484), and monocyte chemotactic protein-1 (MCP-1, JL20304) in the kidney were also examined by ELISA kits (Shanghai Jianglai Industrial Limited by Share Ltd., China), respectively. All experimental procedures were carried out following the manufacturer's instructions.

### 2.5. Histological Assessment

The kidney tissues stored in 10% formalin solution were fixed with 4% paraformaldehyde and then dehydrated, embedded in paraffin wax, and sectioned into 4 *μ*m slices. The sections were stained with hematoxylineosin (H&E), periodic acid-Schiff (PAS), and Masson's trichrome (Masson). The morphology of kidney tissues was observed using a light microscope for histopathological analysis. H&E staining results were reported descriptively. The glomerular injury was assessed by PAS and calculated from the integrated optical density (IOD), which is also equal to the positive area × average density. On the basis of Masson staining, tubulointerstitial fibrosis was assessed by the percentage of collagen area: collagen area (%) = the positive area/total area × 100.

### 2.6. Detection of ROS

ROS production in the kidneys was further detected using DHE staining. Briefly, 10 *μ*m thick frozen renal tissues were obtained using a freezing microtome (CM1900, Leica, Germany) and samples were incubated with PBS for 15 min. Subsequently, tissues were incubated with 5 mmol/L fluorescent-labeled DHE which was diluted by 1 : 1000 (KGAF019, KeyGEN BioTECH, Nanjing, China) in a lucifugal humidified chamber at 37°C for 30 min, and then were stained with 4′,6′-diamidino-2-pheny-lindole (DAPI, AS1075, Aspen Biological, Wuhan, China). Images were taken at 400x magnification under a fluorescence microscope (MicroPublisher, MP3.3-RTV-CLR-10, Q-IMAGING, Canada). The quantitative analysis of the average DHE fluorescence intensity was measured by Image-Pro Plus 6.0 (IPP, Media Cybernetics, Rockville, MD, USA). Results were expressed by the ratio of the fluorescence intensity of DHE-positive area to the DAPI.

### 2.7. Immunofluorescence Imaging

Immunofluorescence staining of renal tissues was conducted to observe the extent of EMT. Frozen sections were placed in acetone ethanol and fixed for 20 min and then washed with PBS three times. Next, sections were placed in a 3% solution of hydrogen peroxide and incubated in dark for 10 min at room temperature. These were then washed with PBS for three times and dried with 5% bovine serum albumin (BSA) for 20 min. Samples were incubated with mouse anti-*α*-SMA antibody (BM0002, 1 : 300, Boster) and mouse anti-E-cadherin antibody (GB12082, 1 : 150, Serviceio) at 4°C overnight, and then, goat anti-mouse antibodies were added for 1 h. Subsequently, DAPI was applied for 5 min. Stained sections were imaged at 400x magnification under a fluorescence microscope (MicroPublisher, MP3.3-RTV-CLR-10, Q-IMAGING, Canada). The further quantitative analysis of *α*-SMA and E-cadherin was performed by IPP.

### 2.8. Cell Culture and Treatment

Human proximal tubular epithelial cell lines (HK-2 cells) were purchased from Nanjing Kaiji Biotechnology Development Co., Ltd. (Nanjing, Jiangsu, China) and cultured at 37°C in 5% humidified CO_2_ in Dulbecco's modified Eagle medium (DMEM)/Ham's F12 media (1 : 1, Gibco, Grand Island, NY, USA) containing 10% fetal bovine serum, 100 U/mL penicillin, and 100 *μ*g/mL streptomycin (Beyotime, Nanjing, Jiangsu, China). The medium was changed every five days. HK-2 cells were cultured in 6-well plates and divided into four experimental groups: Group A: HK-2 cells were treated with 5.5 mM D-glucose; Group B: HK-2 cells were treated with concentrations of 30 mM D-glucose; Group C: HK-2 cell was treated with 30 mM D-glucose plus 0.4 mg/mL YSPDF; and Group D: HK-2 cells were treated with 30 mM D-glucose plus 0.4 mg/mL YSPDF and 10 *μ*mol Nrf2 inhibitor (ML385). After a 48 h incubation, the cells were lysed in M-PER mammalian protein extraction reagent (Thermo Fisher) for western blotting analysis.

### 2.9. Western Blot

Western blots were performed as previously described [25]. Total protein was extracted from kidney tissue in RIPA lysis buffer (25 mM Tris-HCl, 25 mM NaCl, 0.5 mM EDTA, 1% Triton X-100, and 0.1% SDS) with 1% PMSF protease inhibitors (P1005, Beyotime Biotechnology, China) and phosphatase inhibitors (P1081, Beyotime Biotechnology, China) added. Equal amounts of protein were separated by 10-15% SDS-PAGE and then transferred to PVDF membranes (IPVH00010, Millipore, Germany). After blocking with 5% nonfat milk for 3 h, the membranes were incubated with the primary antibodies including TGF-*β*1, *α*-SMA, Nrf2, HO-1, NQO1, ASC, caspase-1 (ab215715, ab32575, ab137550, ab13248, ab80588, ab175449, ab1872, Abcam, UK), p-Smad2, Smad2, p-Smad3, Smad3, E-cadherin, NLRP3 ((#18338, #5339, #9520, #9523, #3195, #15101, Cell Signaling Technology, USA), and cleaved caspase-1 (AF4005, Affinity Biosciences, USA) at 4°C overnight. Then, the membranes were washed 3 times with TBST and incubated with the secondary antibodies for 1 h at room temperature. After washing with TBST for a further three times, the protein bands were visualized by enhanced chemiluminescence (32134, Thermo, USA) solution and imaged with Automated Imaging System (Gene Gnome5, Synoptics Ltd, UK). GAPDH or *β*-actin was assumed to be similarly abundant in all samples and was used as a loading control.

### 2.10. Statistical Analysis

Statistical analysis was performed using SPSS 22.0 software (IBM Corporation, Armonk, New York, USA). All experimental data were expressed as the mean ± SEM. Differences were determined by one-way ANOVA test and Dunnett's test. *p* values less than 0.05 were considered as statistically significant.

## 3. Results

### 3.1. Quality Analysis of YSPDF

**Figure 1:**
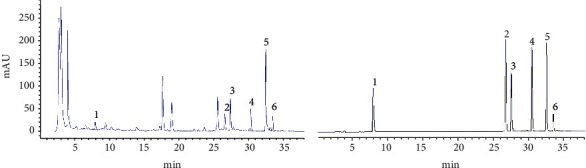
(a) HPLC chromatogram of YSPDF: (1) calycosin glucoside, (2) aloe-emodin emodin, (3) rhein, (4) emodin, (5) chrysophanol, and (6) physcion. (b) HPLC chromatogram of standard solution: (1) calycosin glucoside, (2) aloe-emodin emodin, (3) rhein, (4) emodin, (5) chrysophanol, and (6) physcion.

The aqueous extract of the mixture containing four Chinese medicinal materials was measured by HPLC. Six compounds 1-6, with the retention time at 7.96 min, 26.81 min, 27.56 min, 30.40 min, 32.74 min, and 33.51 min, respectively, were identified as (1) calycosin glucoside, (2) aloe-emodin emodin, (3) rhein, (4) emodin, (5) chrysophanol, and (6) physcion according to results of the HPLC chromatogram of YSPDF (Figure 1). The ratio of the six identified compounds was approximately 1 : 1.9 : 3.2 : 2 : 7.5 : 1.2.

### 3.2. YSPDF Improved Blood and Urine Biochemical Parameters in *db/db* Mice

As shown in Table 2, the levels of FBG and the kidney index of the untreated *db/db* mice were significantly increased compared with the control group, while the *db/db* mice receiving YSPDF treatment were significantly reduced. However, there were no significant changes in body weight. Results indicated that YSPDF was able to reduce the fasting blood glucose levels of *db/db* mice and potentially might have a protective effect on kidney.

Moreover, higher levels of SCr and 24 h UTP and lower levels of urine creatinine and creatinine clearance in *db/db* mice were observed compared with control mice. The levels of serum TC, TG, and LDL were also markedly elevated in the untreated DKD group, while HDL level was decreased, demonstrating an impairment in renal function. In contrast, treatment with YSPDF significantly reduced SCr, 24 h UTP, TC and TG levels compared to the untreated DKD group. YSPDF treatment also increased the levels of urine creatinine, creatinine clearance, and HDL of DKD mice. These above results demonstrated that YSPDF may not only prevent kidney damage and improve renal function but also reduce levels of blood lipids.

### 3.3. YSPDF Alleviated the Histopathological Changes of Kidney in *db/db* Mice

**Figure 2:**
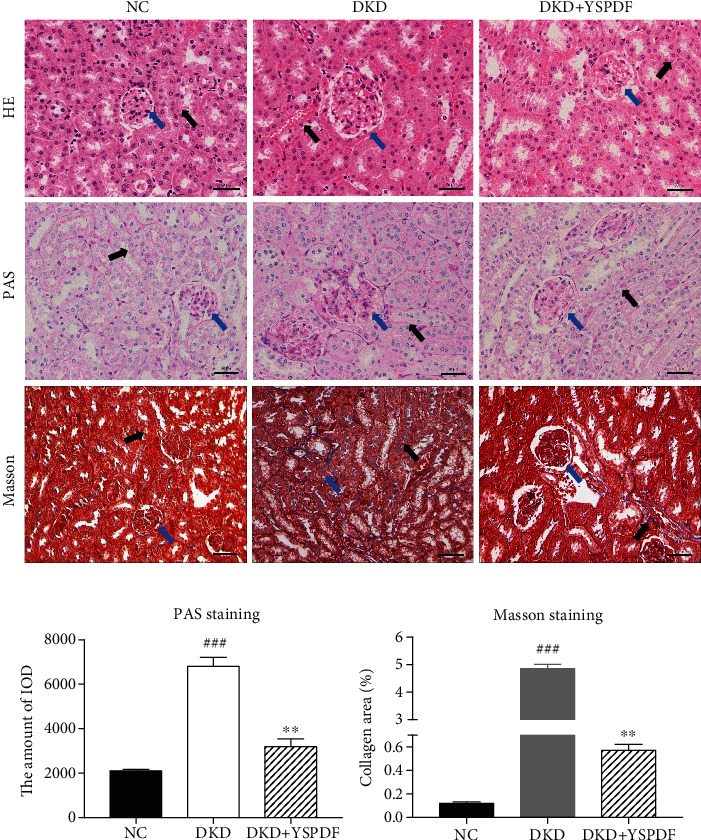
Effect of YSPDF on histopathological changes of the kidney in *db/db* mice. (a) Representative images of H&E, PAS, and Masson staining of kidney tissues were shown (400x). Each bar indicates 50 *μ*m. (b) Glomerular injury using PAS staining and tubulointerstitial fibrosis using Masson staining. Blue arrows show the glomeruli, and the black arrows show kidney tubules. Data were presented as the mean ± SEM; *n* = 3; ^###^*p* < 0.001 compared with the normal control group; ^∗∗^*p* < 0.01 compared with the DKD group.

The histopathological alterations were investigated in renal tissues via H&E staining, PAS staining, and Masson staining. Generally, H&E staining was used to observe the lesions in kidney tissues, PAS staining was used to examine the glomerular sclerosis index, and Masson staining was used to assess the renal fibrosis. As shown with H&E- and PAS-stained kidney tissues (Figure 2(a), 2(b)), compared with the control group, the untreated DKD group exhibited kidney damage, including abnormal glomerular structure, interstitial edema and hyperemia, thickening of the glomerular basement membrane, and an increase in the amount of inflammatory cell infiltration and fibrosis. In contrast, glomerular injury, renal damage and inflammatory responses were significantly reduced by YSPDF treatment.

Masson staining (Figures 2(a) and 2(b)) showed that the basement membrane of renal tissue in DKD mice was much bluer, and the distribution of Masson-positive areas was significantly enlarged, indicating the increasing content of collagen fibers and collagen deposition, signs of fibrosis. These phenomena were significantly improved after treatment with YSPDF. Results indicated that YSPDF could ameliorate tubulointerstitial damage, renal fibrosis in DKD mice compared with untreated animals.

### 3.4. YSPDF Mitigated Renal Fibrosis in Kidney of *db/db* Mice by Inhibiting EMT

**Figure 3:**
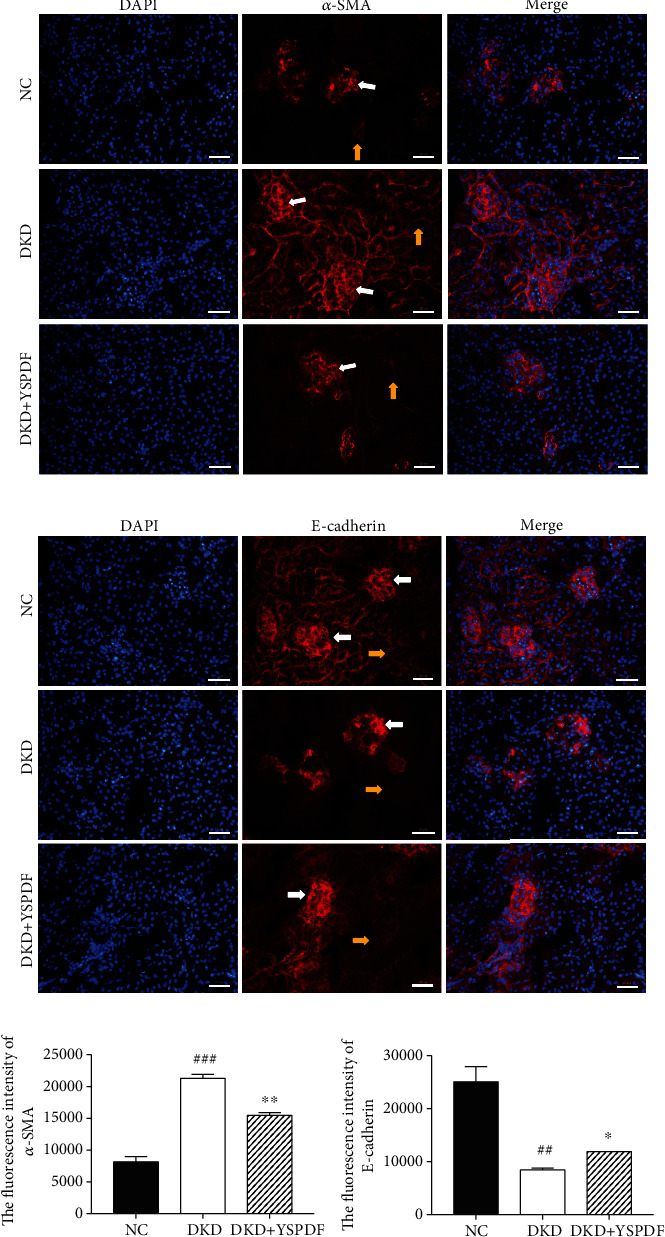
Representative immunofluorescence images of (a) *α*-SMA (red) and (b) E-cadherin (red). Each bar indicates 50 *μ*m. (c) The fluorescence intensity was measured by IPP, and results were expressed by the ratio of fluorescence intensity of the positive area to DAPI. White arrows show the glomeruli, and the yellow arrows show kidney tubules. Data were presented as the mean ± SEM; *n* = 3; ^##^*p* < 0.01 and ^###^*p* < 0.001 compared with the normal control group; ^∗^*p* < 0.05 and ^∗∗^*p* < 0.01 compared with the DKD group.

To estimate whether YSPDF could reduce EMT in the kidneys of db/db mice, we determined the levels of EMT-associated proteins. Immunofluorescence imaging shows the protein expression (Figure 3(a)) and fluorescence intensity (Figure 3(c)) of *α*-SMA was considerably increased in the kidneys of untreated DKD mice, and this was attenuated with YSPDF treatment. Further results showed that YSPDF administration markedly inhibited E-cadherin expression as compared to untreated DKD mice (Figure 3(b)). The fluorescence intensity of E-cadherin (Figure 3(c)) was reduced in untreated DKD mice, with YSPDF treatment decreasing the amount of this reduction. These results indicated that YSPDF administration might inhibit renal fibrosis by inhibiting EMT.

### 3.5. YSPDF Inhibited Renal Oxidative Stress in *db/db* Mice

**Figure 4:**
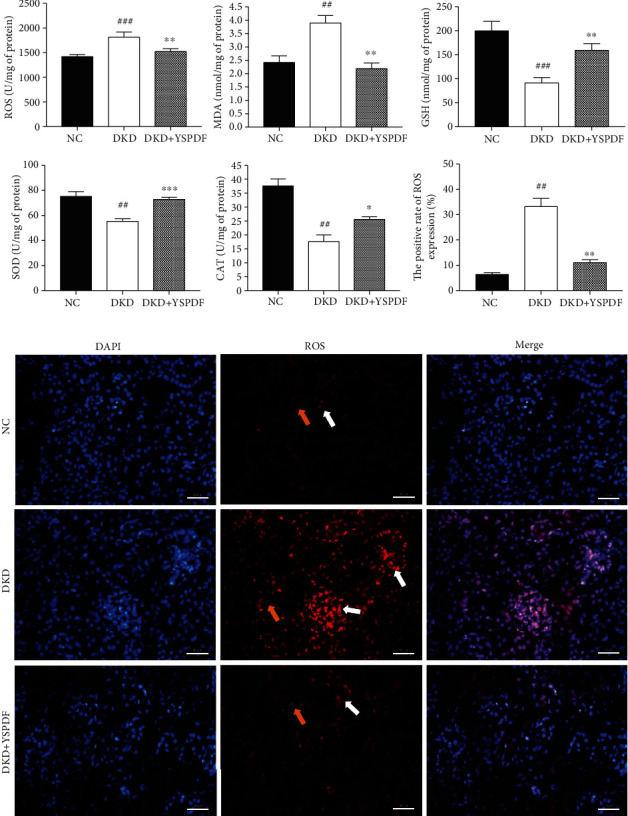
YSPDF ameliorated oxidative stress in *db/db* mice. (a) Oxidative stress parameters including ROS, MDA, GSH, SOD, and CAT were determined by commercial assay kits. DHE fluorescence intensity was measured by IPP, and results were expressed by the ratio of fluorescence intensity of the DHE-positive area to the DAPI. (b) ROS level was determined by DHE staining (400x). Each bar indicates 50 *μ*m. White arrows show the glomeruli, and the yellow arrows show kidney tubules. ^##^*p* < 0.01 and ^###^*p* < 0.001 compared with the normal control group; ^∗^*p* < 0.05, ^∗∗^*p* < 0.01, and ^∗∗∗^*p* < 0.001 compared with the DKD group.

Oxidative stress has been shown to play a crucial role in diabetes-induced kidney injury [8]. Therefore, indicators of oxidative stress injury including ROS level, MDA level, GSH level, and SOD and CAT activities in kidney tissues were measured. As shown in Figure 4(a), levels of ROS and MDA in untreated *db/db* mice were significantly elevated compared with the control group (*p* < 0.01), with this elevation being inhibited by YSPDF treatment. In contrast, the levels of GSH, SOD, and CAT were markedly decreased (*p* < 0.01). Treatment with YSPDF attenuated this decrease for all three parameters. DHE staining showed that YSPDF significantly inhibited ROS production in the kidneys of *db/db* mice as compared with the untreated DKD group mice (Figure 4(b)).

### 3.6. YSPDF Alleviated the Production of Inflammatory Cytokines in the Kidneys of *db/db* Mice

**Figure 5:**
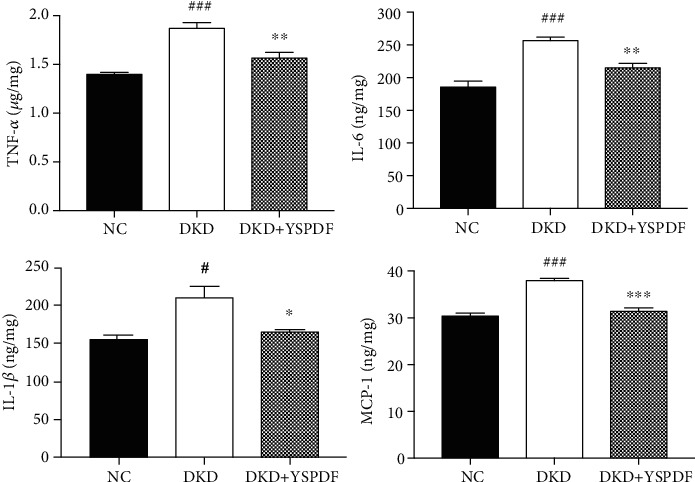
YSPDF inhibited the production of inflammatory factors in *db/db* mice. Proinflammatory factors including TNF-*α*, IL-6, IL-1*β*, and MCP-1 were detected by ELISA assay. ^#^*p* < 0.05 and ^###^*p* < 0.001 compared with the normal control group; ^∗^*p* < 0.05, ^∗∗^*p* < 0.01, and ^∗∗∗^*p* < 0.001 compared with the DKD group.

To confirm the effect of YSPDF on inflammation and renal damage, the expressions of proinflammatory cytokines including TNF-*α*, IL-6, IL-1*β*, and MCP-1 were measured. ELISA assays (Figure 5) showed that the levels of TNF-*α*, IL-6, IL-1*β*, and MCP-1 in the kidney tissues of DKD mice were significantly higher than those of control mice. YSPDF treatment significantly decreased the levels of these inflammatory factors.

### 3.7. YSPDF Treatment Inhibited Renal Fibrosis in the Kidneys of *db/db* Mice by Blocking EMT via Suppressing the TGF-*β*1/Smad2/3 Signaling Pathway

**Figure 6:**
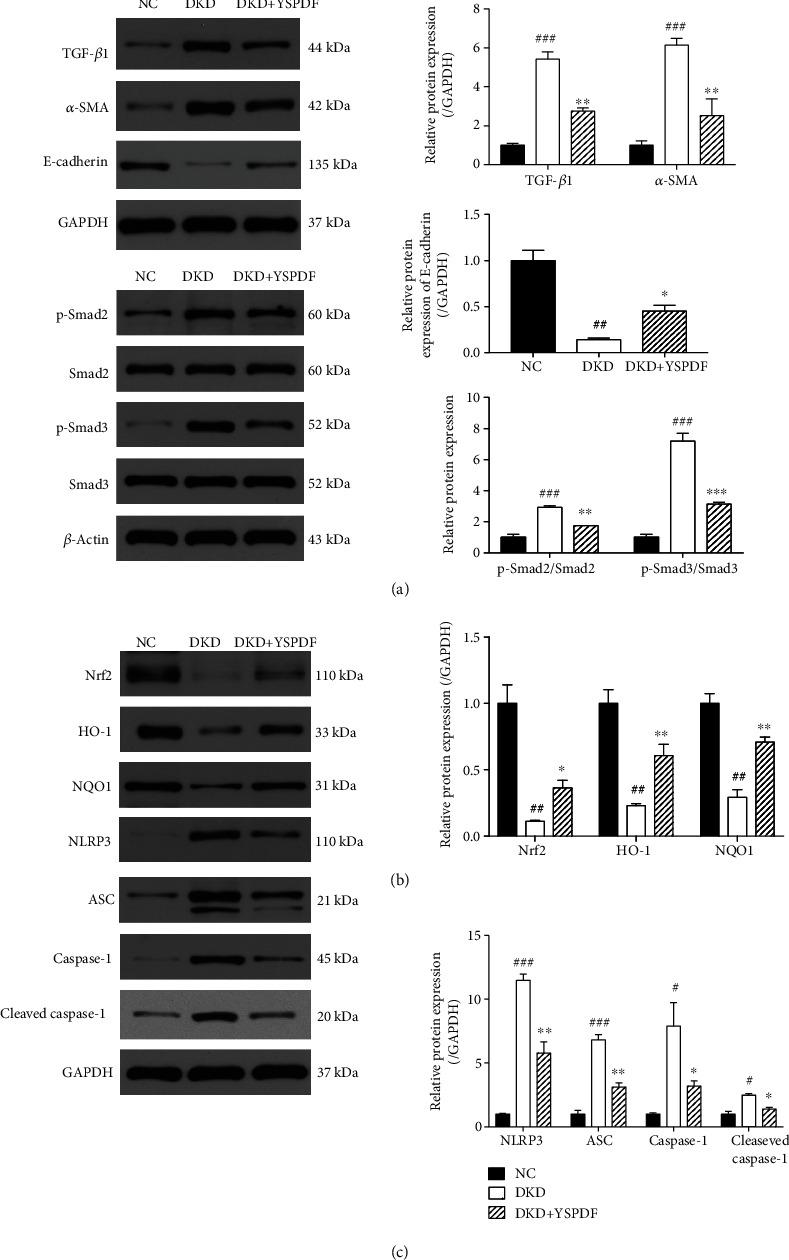
YSPDF treatment regulated the expressions of TGF-*β*1, p-Smad2, Smad2, p-Smad3, Smad3, *α*-SMA, E-cadherin, Nrf2, HO-1, NQO1, NLRP3, ASC, caspase-1, and cleaved caspase-1 in the kidneys of *db/db* mice. Protein expression levels were normalized to the levels of either GAPDH or *β*-actin. The data were analyzed using a one-way ANOVA and expressed as the mean ± SEM (*n* = 3). ^#^*p* < 0.05, ^##^*p* < 0.01, and ^###^*p* < 0.001 compared with the normal control group; ^∗^*p* < 0.05 and ^∗∗^*p* < 0.01 compared with the DKD group.

TGF-*β*1 has been shown to play a crucial role in the development of renal cell hypertrophy and tubulointerstitial fibrosis via its downstream Smad-dependent pathway and the regulation of the expression of EMT proteins such as *α*-SMA and E-cadherin [20]. As indicated in Figure 6(a), the expression levels of TGF-*β*1, p-Smad2, p-Smad3, and *α*-SMA were markedly increased in the kidney tissues of untreated DKD mice as compared to the control group. However, the expression levels of these proteins were significantly suppressed by YSPDF. Additionally, the level of E-cadherin expression was downregulated in the untreated DKD group, which was partially recovered by YSPDF treatment. Therefore, our results further indicated that YSPDF may suppress the progression of renal fibrosis via inhibiting EMT by blocking the TGF-*β*1 signaling pathway.

### 3.8. YSPDF Protected Kidneys from Injury by Activating Nrf2 and Inhibiting NLRP3 Inflammasome Signaling Pathways in *db/db* Mice

We investigated further the underlying molecular mechanism explaining the protection of YSPDF on DKD by analyzing its effects on the important antioxidant signaling Nrf2 pathway. The key proteins of the Nrf2 pathway, including Nrf2, HO-1 and NQO1, were examined by western blot. The expressions of Nrf2 and its downstream factors HO-1 and NQO1 were distinctly reduced in untreated DKD mice (Figure 6(b)). Treatment with YSPDF partially reversed these changes. We also evaluated the effect of YSPDF on the inhibition of the renal NLRP3 inflammasome, which has been reported to be important in the development of renal injury [12]. Western blot results showed that the expression levels of inflammasome proteins including NLRP3, ASC, caspase-1, and cleaved caspase-1 in the kidneys of DKD mice were significantly elevated compared with the control group (Figure 6(c)). In contrast, YSPDF partially rescued the levels of NLRP3, ASC, caspase-1, and cleaved caspase-1 when compared to the untreated DKD group. Collectively, these results demonstrated that YSPDF might ameliorate oxidative stress and inflammation by activating the Nrf2 pathway and by inhibiting the NLRP3 inflammasome pathway.

### 3.9. YSPDF Inhibited EMT and NLRP3 Inflammasome Signaling Pathway by Activating Nrf2 Pathway in High Glucose- (HG-) Induced HK-2 Cells

**Figure 7:**
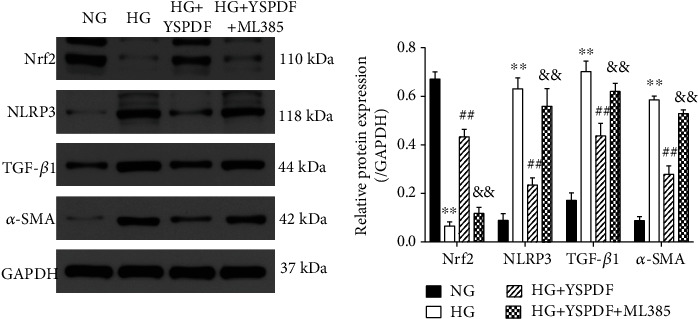
The expressions of Nrf2, NLRP3, TGF-*β*1, and *α*-SMA in HK-2 cells treated by NG, HG, HG+YSPDF, and HG+YSPDF+ML385. Protein expression levels were normalized to the levels of GAPDH. The data were analyzed using a one-way ANOVA and expressed as the mean ± SEM (*n* = 3). ^∗∗^*p* < 0.01 compared with the NG group; ^##^*p* < 0.01 compared with HG group; ^&&^*p* < 0.01 compared with the HG+YSPDF group.

To support the previous in vivo results, HK-2 cells in HG conditions were treated with YSPDF (Figure 7). Results demonstrated that HG significantly increased the expressions of *α*-SMA, TGF-*β*1, and NLRP3 and decreased the expression of Nrf2 in HK-2 cells as compared to the NC group (*p* < 0.01). Nevertheless, compared with the HG group, *α*-SMA, TGF-*β*1, and NLRP3 expressions in the YSPDF group were decreased, whereas that of Nrf2 was increased. Next, to determine whether Nrf2 was required for YSPDF protection from HG-induced harmful effects, a Nrf2 inhibitor (ML 385) was used to inhibit Nrf2 expression in HK-2 cells. As shown in Figure 7, ML385 reduced Nrf2 expression in YSPDF-treated cells, which resulted in increasing expressions of *α*-SMA, TGF-*β*1, and NLRP3. This result suggested that YSPDF-induced activation of the Nrf2 pathway was prevented by the Nrf2 inhibitor. Accordingly, YSPDF induced inhibition of the NLRP3 inflammasome pathway, TGF-*β*1 pathway, and EMT were partly reversed in HG-exposed HK-2 cells treated with ML385. These findings suggested that YSPDF protected HK-2 cells from HG-induced injury in a manner dependent on Nrf2 activation.

## 4. Discussion

The pathogenesis of DKD is complicated and accumulating studies show that oxidative stress, inflammation, and EMT played important roles in disease progression, in addition to a disorder in blood glucose and lipid metabolism [26–28]. Consequently, revealing more information on the mechanisms behind kidney injury in DKD and finding new drugs or therapeutic targets has become a research hotspot globally.

In recent years, traditional Chinese medicine (TCM) has shown broad and promising application prospects in the treatment of DKD due to its advantage of having low toxicology and high efficiency [3, 29]. YSPDF has been used in clinical practice in our hospital for twenty years with no reports of significant side effects [24]. Results showed that YSPDF, coated aldehyde oxystarch capsules and a combination therapy of YSPDF with coated aldehyde oxystarch capsules could reduce SCr and uric acid, increase eGFR, and protect renal function in patients with CKD3 and CKD4, with the cotreated group being more significant than in the YSPDF and coated aldehyde oxystarch capsule-treated groups [30]. The acute toxicity assay results of YSPDF in healthy Sprague Dawley (SD) rats showed that none of the rats died and the visceral organs had no changes in gross anatomy. Histopathological analysis of the liver and kidney showed no toxicity in the experimental animals, and no significant changes were found in biochemical parameters (including white blood cell count, red white blood cell count, hemoglobin, platelet count, alamine aminotransferase, aspartate transaminase, serum creatinine, and urea nitrogen) when compared to those in the control group [24]. All results suggest that YSPDF is safe and effective and will not cause renal function damage.

In the present study, we used an experimental animal model of DKD using *db/db* mice. A significant increase of the kidney index, FBG, SCr, 24 h UTP, serum cholesterols, and triglycerides were observed in DKD mice, which is consistent with previous studies [14, 31]. Several clinical studies have shown that long-term hyperglycemia was the most critical element that mediated the progressive tissue damage and functional decline characterizing diabetic complications in DKD [14, 31]. Long-term hyperglycemia induces insulin resistance and oxidative stress. Insulin resistance activates signaling pathways, such as P38 and extracellular signal-regulated kinase (ERK) pathways, to increase the expression of TGF-*β*, while increased oxidative stress cooperates with the activated polyol pathway to stimulate the protein kinase C (PKC) pathway [14, 31]. All of these contribute to the deposition of ECM and leads to glomerulosclerosis and tubular interstitial fibrosis. Thus, the controlling of blood glucose levels could effectively delay the progression of DKD [14, 31]. Here, we find that YSPDF was able to significantly decrease blood glucose levels in *db/db* mice, which indicates that the protective effect of YSPDF might be related its hypoglycemic activity. Moreover, histopathological results showed renal fibrosis as well as accumulation of inflammatory cell infiltration in the untreated DKD mice. On the contrary, YSPDF treatment not only partially restored blood and urine parameters but also helped ameliorate the pathological changes in the kidneys of the diabetic mice. These results demonstrated that YSPDF was an effective treatment for protecting against and alleviating renal damage and renal fibrosis.

TGF-*β*1/Smad signaling is currently recognized as one of the most potent profibrogenic factors and has been shown to be a crucial mediator in EMT [32, 33]. Increased expression of *α*-SMA and a decreased expression of E-cadherin were major hallmarks of EMT. TGF-*β*1 is able to activate Smad2/3 via phosphorylation, accelerating the process of fibrosis [34]. In this study, the expression levels of TGF-*β*1, p-Smad2/Smad2, p-Smad3/Smad3, and *α*-SMA were significantly upregulated in untreated *db/db* kidney tissues, with a significant dampening of this affect seen in the YSPDF-treated group. Moreover, YSPDF significantly increased the expression of E-cadherin compared to the untreated *db/db* mice from the results of western blot and immunofluorescence assay. Our results were consistent with a previous observation that TGF-*β*1 might induce EMT and ROS generation, where it was shown that treatment with an antioxidant was able to markedly prevent EMT in HK-2 cells [32]. These experimental results suggest that inhibition of tubular EMT to mitigate renal fibrosis via inhibiting the TGF-*β*1/Smad signaling pathway might be one of the mechanisms by which YSPDF was able to alleviate kidney damage.

The excessive production of ROS in renal cells under the condition of hyperglycemia is considered an important pathological mechanism for DKD [31]. In the present study, our results found that the levels of GSH, SOD, and CAT activities were downregulated in the kidneys of untreated DKD mice, whereas ROS and MDA increased, demonstrating severe oxidative damage occurring in the kidneys of *db/db* mice. YSPDF treatment partially reversed these changes and distinctly suppressed ROS production, implying that the renal protective effect of YSPDF might be associated with its strong antioxidative properties.

Nrf2 is a critical redox-sensitive transcription factor, and once activated, it transfers to the nucleus and activates the transcription of many antioxidant genes, such as HO-1 and NQO1, thus reducing oxidative stress [35–37]. It was reported that Nrf2/HO-1 was able to inhibit the pathological process of DKD by decreasing oxidative stress and inflammation [38]. Our findings were consistent with those of other studies showing that the expression levels of Nrf2, and its downstream targets HO-1 and NQO1 were significantly decreased in the untreated DKD group compared with the nondiabetic control group. This effect was significantly attenuated with YSPDF administration, indicating that the Nrf2 pathway may be involved in the antioxidative and protective effect of YSPDF against DKD. Previous research has reported that treatment of a rat glomerular mesangial cell line (HBZY-1) with an Nrf2 inhibitor (ML385) suppressed the activation of Nrf2, HO-1, and NQO1 and that this abolished the protective effect of treatments reducing oxidative stress [39]. Similarly, we showed that YSPDF activated Nrf2 in HG-induced HK-2 cells, which resulted in the reductions of *α*-SMA and TGF-*β*1 expression. Nevertheless, YSPDF-mediated activation of the Nrf2 pathway was further inhibited by ML385. These findings support the hypothesis that YSPDF ameliorates renal damage in DKD mice and HG-exposed HK-2 cells by activating the Nrf2 pathway.

There is evidence that hyperglycemia increases the release of proinflammatory cytokines and promotes the synthesis of ECM proteins in glomeruli cells, leading to renal inflammation and fibrosis [39]. Several proinflammatory cytokines, such as TNF-*α*, IL-6, IL-1*β*, and IL-18 were reported to participate in the pathogenesis of DKD [39]. In this study, *db/db* mice had increased levels of TNF-*α*, IL-6, IL-1*β*, and MCP-1 in kidney tissues, consistent with published studies [38, 40]. YSPDF treatment helped reverse this increase, suggesting its inhibitory effects on inflammation.

Accumulating studies have shown that the NLRP3 inflammasome, which is composed of the NLRP3 protein, caspase-1, and ASC, plays a pivotal role in DKD kidney inflammation, especially in podocytes, endothelial cells, and renal tubular epithelial cells [41, 42]. Once cells were subjected to stress, NLRP3 quickly activated and recruited ASC and pro-caspase-1, leading to activation of caspase-1, thereby resulting in the secretion of pro-inflammation cytokines such as IL-1*β* and IL-18 in DKD [28, 43]. Moreover, oxidative stress was also reported to be the key element of activating the NLRP3 inflammasome [44, 45]. ROS-induced NLRP3 activation was shown to accelerate the kidney damage in mice fed a high-fat diet [45].

According to our western blot and ELISA assay results, expression levels of NLRP3, ASC, and caspase-1 proteins and IL-1*β* in the kidneys of DKD mice were markedly increased. Furthermore, an abnormal glomerular structure and a large amount of inflammatory cell infiltration and fibrosis were also observed in the DKD group. However, these phenomena were significantly normalized in mice treated with YSPDF. In line with our findings, NLRP3 deficiency has been shown to mitigate renal inflammation and fibrosis in DKD [15]. Inhibition of NLRP3 inflammasome activation by silencing NLRP3/ASC or repressing caspase-1 activity has been shown to decrease the production of IL-1*β* and alleviate kidney damage [16]. Several lines of evidence have revealed that diabetes-induced renal NLRP3 inflammasome activation, TGF-*β*1/Smad3 activation, and ECM protein accumulation were inhibited in NLRP3 knockout mice [15]. In addition, Nrf2 activation could also inhibit the NLRP3 pathway. In this study, treatment with YSPDF significantly increased Nrf2 expression and reduced NLRP3 expression in HG-exposed HK-2 cells, whereas this effect of YSPDF was abolished by a Nrf2 inhibitor. Therefore, we suggest that YSPDF may protect kidneys from damage in DKD via suppression of the production of inflammatory cytokines and by inhibiting the activation of the NLRP3 inflammasome via activating the Nrf2 pathway.

## 5. Conclusion

**Figure 8:**
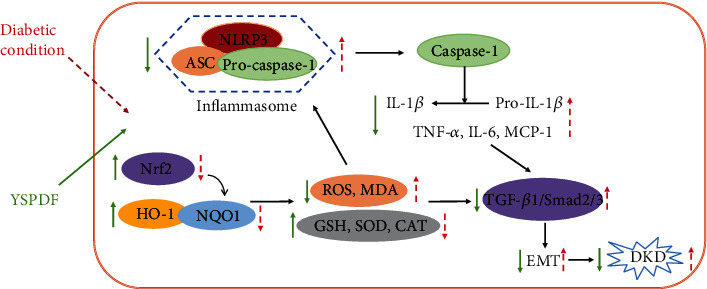
The possible molecular mechanisms of YSPDF on DKD.

In conclusion, as a traditional Chinese medicine preparation, YSPDF exhibited a renal protective effect in *db/db* mice. Moreover, we demonstrated that YSPDF can attenuate oxidative stress and inflammatory states in the kidneys of DKD mice via activating the Nrf2 pathway, inhibiting the NLRP3 inflammasome pathway, and reducing renal tubular EMT via inhibiting the TGF-*β*1/Smad pathway, thus reducing kidney injury. Cell experiments showed that YSPDF inhibited EMT and the NLRP3 inflammasome in HG-exposed HK-2 cells, possibly through activation of Nrf2. The possible mechanisms of YSPDF treatment of DKD were summarized in Figure 8. Therefore, we conclude that YSPDF may be a potential and promising drug to treat DKD.

## Figures and Tables

**Table 1:** The components and ratio of YSPDF.

Pharmaceutical name	Botanical plant name/animal medicinal materials animal name	Family	Medicinal part	Ratio
*Astragali Radix*	*Astragalus membranaceus (Fisch). Bge.* var. *mongholicus (Bge). Hsiao*	Leguminosae	Root	25
*Rhei radix et rhizoma*	*Rheum palmatum L.*	Polygonaceae	Root and rhizome	5
*Hirudo*	*Whitmania pigra Whitman*	Hirudinidae	Drying body	3
*Bombyx batrytocatus*	*Bombyx mori Linnaeus*	Bombycidae	Drying body	3

**Table 2:** YSPDF improved the levels of various biochemical parameters in *db/db* mice.

Biochemical parameters	NC	DKD	DKD+YSPDF
Final body weight (g)	29.28 ± 2.74	46.42 ± 5.31^###^	43.76 ± 3.21
Kidney index (%)	0.96 ± 0.11	1.33 ± 0.32^##^	1.07 ± 0.42^∗^
Fasting blood glucose (mmol/L)	4.42 ± 0.74	23.78 ± 5.61^###^	15.26 ± 4.64^∗∗^
Serum creatinine (*μ*mol/L)	2.59 ± 0.41	5.52 ± 0.51^###^	2.98 ± 0.87^∗∗∗^
Urea nitrogen (mmol/L)	6.01 ± 0.14	18.29 ± 4.11^###^	9.36 ± 1.83^∗∗^
24 h urine protein (mg/L)	1.37 ± 0.36	5.87 ± 1.54^###^	1.96 ± 0.87^∗∗∗^
Urine creatinine (mmol/L)	2.42 ± 0.56	0.36 ± 0.12^###^	0.81 ± 0.31^∗∗∗^
Creatinine clearance (mL/min)	1.82 ± 0.62	0.93 ± 0.15^##^	1.46 ± 0.77^∗∗^
Total cholesterol (mmol/L)	3.86 ± 0.44	7.03 ± 0.91^###^	4.66 ± 1.09^∗∗∗^
Triglyceride (mmol/L)	1.14 ± 0.41	3.68 ± 0.79^###^	2.43 ± 0.49^∗^
HDL (mmol/L)	1.77 ± 0.35	1.07 ± 0.38^#^	2.25 ± 0.75^∗^
LDL (mmol/L)	0.30 ± 0.11	0.51 ± 0.15^#^	0.59 ± 0.47

NC refers to the normal control group; DKD refers to diabetic kidney disease group. Data were expressed as the mean ± SEM (*n* = 8). ^#^*p* < 0.05, ^##^*p* < 0.01, and ^###^*p* < 0.001 compared with the normal control group; ^∗^*p* < 0.05, ^∗∗^*p* < 0.01, and ^∗∗∗^*p* < 0.001 compared with the DKD group.

## Data Availability

The data used to support the findings of this study are available from the corresponding author upon request.

## Abbreviations

YSPDF: Yi Shen Pai Du formula
DKD: Diabetic kidney disease
CKD: Chronic kidney disease
EMT: Epithelial-to-mesenchymal transition
DN: Diabetic nephropathy
ESRD: End-stage renal failure
ECM: Extracellular matrix
TGF-*β*1: Transforming growth factor-*β*1
*α*-SMA: *α*-Smooth muscle actin
Nrf2: Nuclear factor-erythroid 2-related factor 2
NLRP3: Nucleotide binding and oligomerization domain-like receptor family pyrin domain-containing 3
ASC: Apoptosis-associated speck-like protein containing a caspase recruitment domain
ROS: Reactive oxygen species
RP-HPLC: Reversed phase high-performance liquid chromatography
UCr: Urinary creatinine
FBG: Fasting blood glucose
SCr: Serum creatinine
BUN: Blood urea nitrogen
TC: Total cholesterol
TG: Total triglycerides
HDL: High-density lipoprotein
LDL: Low-density lipoprotein
GSH: Glutathione
MDA: Malondialdehyde
SOD: Superoxide dismutase
ELISA: Enzyme-linked immunosorbent assay
IL-1*β*: Interleukin-1*β*
IL-6: Interleukin-6
TNF-*α*: Tumor necrosis factor-*α*
MCP-1: Monocyte chemotactic protein-1
HE: Hematoxylineosin
PAS: Periodic acid-Schiff
DAPI: 4′,6′-Diamidino-2-pheny-lindole.
